# The Unfolded Protein Response in Breast Cancer

**DOI:** 10.3390/cancers10100344

**Published:** 2018-09-21

**Authors:** Eoghan P. McGrath, Susan E. Logue, Katarzyna Mnich, Shane Deegan, Richard Jäger, Adrienne M. Gorman, Afshin Samali

**Affiliations:** 1 Apoptosis Research Centre, National University of Ireland (NUI), Galway, University Road, Galway, H91 TK33 Galway, Ireland; E.MCGRATH14@nuigalway.ie (E.P.M.); susan.logue@nuigalway.ie (S.E.L.); katarzyna.mnich@nuigalway.ie (K.M.); shane.deegan@nuigalway.ie (S.D.); adrienne.gorman@nuigalway.ie (A.M.G.); 2 School of Natural Sciences, NUI Galway, University Road, H91 TK33 Galway, Ireland; 3 Department of Natural Sciences, Bonn-Rhein-Sieg University of Applied Sciences, 53359 Rheinbach, Germany; Richard.Jaeger@h-brs.de

**Keywords:** breast cancer, endoplasmic reticulum (ER) stress, unfolded protein response (UPR), therapy, cell death, autophagy

## Abstract

In 2018, in the US alone, it is estimated that 268,670 people will be diagnosed with breast cancer, and that 41,400 will die from it. Since breast cancers often become resistant to therapies, and certain breast cancers lack therapeutic targets, new approaches are urgently required. A cell-stress response pathway, the unfolded protein response (UPR), has emerged as a promising target for the development of novel breast cancer treatments. This pathway is activated in response to a disturbance in endoplasmic reticulum (ER) homeostasis but has diverse physiological and disease-specific functions. In breast cancer, UPR signalling promotes a malignant phenotype and can confer tumours with resistance to widely used therapies. Here, we review several roles for UPR signalling in breast cancer, highlighting UPR-mediated therapy resistance and the potential for targeting the UPR alone or in combination with existing therapies.

## 1. Introduction

Breast cancer encompasses a heterogeneous set of diseases with distinct prognoses, physiological and histological characteristics, and treatment options [1]. Different breast cancer subtypes are commonly diagnosed based on the histological expression of three receptor proteins: estrogen receptor (ESR1, also known as ERα), progesterone receptor (PGR), and human epidermal growth factor receptor 2 (HER2, also known as ERBB2), and by the differential expression of fifty select genes (PAM50) which infer the “intrinsic” subtype. Subtyping breast cancer based on these parameters informs the clinician on the best course of treatment for the patient and has led to great improvements in survival rates. In PAM50 analyses, tumours with a gene expression profile typical of luminal epithelial cells belong to the luminal subtype (of which there are two sub-categories), and are usually hormone receptor positive (ESR1+ PGR+). Most breast tumours are luminal and are often responsive to ESR1 modulators, like tamoxifen, or aromatase inhibitors such as anastrozole. HER2+ cancers overexpress HER2 and are generally treated with antibodies targeting HER2 alone, or in combination with chemotherapeutics. Tumors exhibiting a myoepithelial PAM50 profile are referred to as basal-like tumours and are usually triple negative breast cancers (TNBC) in that they do not express ESR1 and PGR and do not have amplified HER2 expression. TNBC patients have a relatively poor outcome compared to other subtypes and TNBC currently lacks a targeted therapy [2,3,4].

The current toolbox of therapies available in the clinic has resulted in a high percentage of breast cancer patients going into remission following treatment. Unfortunately, the remission period for many patients is short-lived and is frequently followed by the reappearance of drug-resistant tumour clones. Discovering and targeting mechanisms by which tumours acquire drug resistance is a primary goal for the breast cancer field [5].

The endoplasmic reticulum (ER) is a complex cellular organelle responsible for the folding and post-translational processing of membrane bound and secreted proteins. Disruption of ER homoeostasis can cause misfolded proteins to accumulate within the ER lumen. This condition is known as ER stress and leads to the (normally) transient activation of a cellular stress response referred to as the unfolded protein response (UPR). While the UPR primarily works to reduce the backlog of unfolded proteins and restore ER function, severe or prolonged UPR signals can trigger cell death [6].

**Figure 1:**
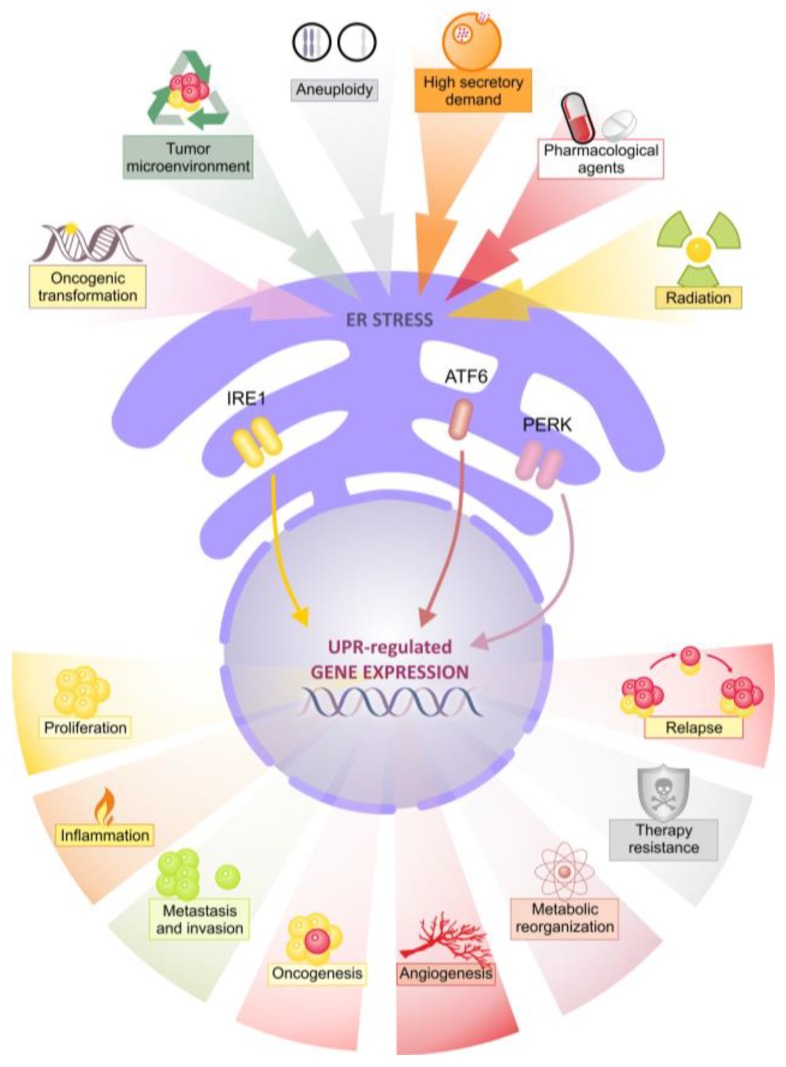
Multiple tumour-associated stressors induce endoplasmic reticulum (ER) stress and pro-tumour unfolded protein response (UPR) signalling. A variety of cell intrinsic and extrinsic stressors lead to UPR activation. In turn the UPR drives multiple pro-tumour processes associated with worse patient outcome.

Within the microenvironment of solid tumours, cancer cells are exposed to a variety of stressors, such as reduced oxygen and energy supply, which can lead to perturbed protein folding in the ER and activation of the UPR. As a result, many tumours display constitutive UPR activation which allows them to adapt and thrive under stressful conditions [7]. In the context of breast cancer, chronic UPR signaling may contribute to most, if not all, hallmarks of cancer [8,9] as well as therapy resistance (see Figure 1). Since the UPR is normally inactive in non-tumour cells but active in tumour cells, it is a strong candidate for the development of novel breast cancer treatments. An added benefit is that the UPR poses distinct therapeutic possibilities for different breast cancer subtypes including TNBC. Therefore, an understanding of UPR biology in oncology and elucidation of the potential for UPR-targeting drugs to improve the treatment of breast cancer is worth exploring. Recent translational UPR research has allowed us to gain insight into how the treatment of different breast cancer subtypes can be improved.

## 2. The Unfolded Protein Reponses

**Figure 2:**
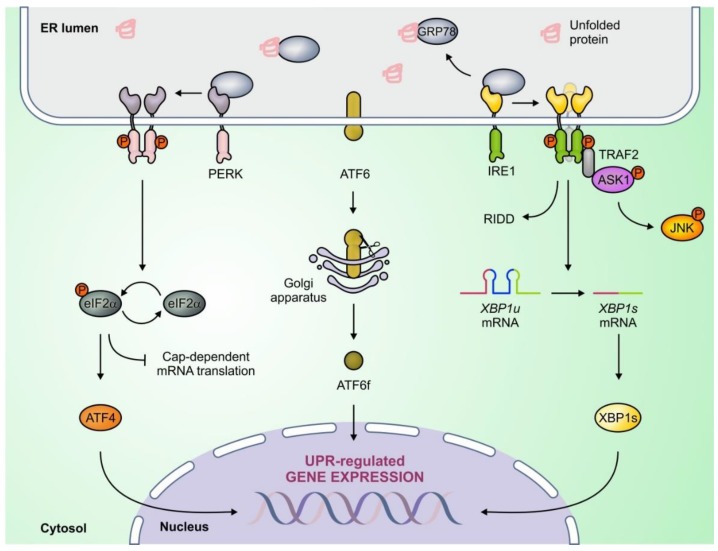
The unfolded protein response (UPR) is mediated by PKR-like ER kinase (PERK), activating transcription factor 6 (ATF6), and inositol-requiring enzyme 1 (IRE1) signaling. Unfolded proteins within the endoplasmic reticulum (ER) lumen lead to activation of ER stress sensors by sequestering glucose-regulated protein 78 kDa (GRP78). ATF4, ATF6f, and spliced x-box binding protein 1 (XBP1s) are adaptive transcription factors activated by the PERK, ATF6, and IRE1 signaling branches respectively, and promote expression of chaperones and protein degradation pathway components. The UPR also engages degradation of cytosolic RNA (regulated IRE1 dependent decay (RIDD) function) and activation of c-Jun N-terminal kinase (JNK) through IRE1, and inhibition of global protein synthesis through PERK.

UPR signalling originates from three sensor proteins which traverse the ER membrane; Inositol Requiring Enzyme 1 (IRE1, also known as ERN1), Activating Transcription Factor 6 (ATF6), and PKR-like ER Kinase (PERK, also known as EIF2AK3). Under non-stressed conditions all three sensors are bound by the chaperone glucose regulated protein 78 kDa (GRP78, also known as BiP or HSPA5) which keeps them in an inactive state. However, upon ER stress, GRP78 detaches from the sensors leading to their activation. The UPR promotes cell survival by driving expansion of the ER, increasing the abundance of protein chaperones (such as GRP78) to help with protein folding, and engaging protein degradation systems, such as autophagy and ER-associated degradation (ERAD). However, if these mechanisms fail to restore homeostasis, the UPR promotes cell death. Temporal regulation of IRE1, PERK, and ATF6 governs the switch between pro-survival and pro-death UPR signalling (See Figure 2) [6,10].

In response to stress, IRE1 can promote either cell-survival or cell death. The N-terminal, ER luminal, region of IRE1 interacts with GRP78 and misfolded proteins within the ER while the cytoplasmic C-terminal possesses both kinase and endoribonuclease (RNase) domains (See Figure 2). Upon ER stress, IRE1 monomers homodimerise and oligomerise leading to juxtaposition of kinase domains, triggering sequential trans-autophosphorylation, a conformational change, and activation of the RNase domain. The IRE1 kinase domain has no reported pro-survival function but can trigger cell death by switching on c-jun n-terminal kinase (JNK) which activates pro-apoptotic BCL-2 family members such as BID and BAD [6,10,11]. The IRE1 RNase domain has two functions, the splicing of X-box binding protein 1 (*XBP1*) mRNA, and Regulated IRE1 Dependent RNA Decay (RIDD) (See Figure 2). IRE1 splices a 26-nucleotide intron from *XBP1* mRNA which is subsequently translated into a transcription factor called spliced XBP1 (XBP1s). XBP1s promotes adaptation to ER stress by upregulating chaperones, the ERAD machinery, and ER expansion-associated genes. The smaller protein encoded by un-spliced *XBP1* mRNA (*XBP1u*) has a short half-life and has been reported to play both agonistic and antagonistic roles in XBP1s signaling [12,13]. IRE1’s RIDD activity involves the selective cleavage of cytoplasmic RNA species by IRE1 and may promote survival by reducing the number of new peptides entering the ER; however RIDD may also promote cell death by cleaving specific miRNA [6,14].

Similar to IRE1, PERK also plays a dual role in cell fate signaling downstream of ER stress. Like IRE1, PERK possesses a cytoplasmic kinase domain that is activated through trans-autophosphorylation [10]. Once activated PERK phosphorylates eukaryotic initiation factor 2α (eIF2α) causing a block in global RNA translation. This block promotes cell survival by lowering the requirement of the ER to fold proteins, and halts cell cycle progression by expediting the depletion of cyclin D1. Furthermore, phosphorylation of eIF2α results in 5’Cap-independent translation of select mRNA, encoding genes such as activating transcription factor 4 (*ATF4*) and its transcriptional targets (See Figure 2) [15]. Some ATF4 target genes, like Autophagy Related 5 (*ATG5*), encode proteins necessary for autophagy, a cell fate-governing mechanism wherein cellular contents are degraded and recycled. Another ATF4 target, C/EBP Homologous Protein (CHOP, also known as DDIT3), promotes death by directly upregulating pro-apoptotic proteins and restoring global protein synthesis through upregulation of GADD34 which dephosphorylates eIF2α [16]. Other direct PERK kinase substrates include nuclear factor erythroid 2-related factor 2 (NRF2) which is part of the anti-oxidant response, the transcription factor FOXO, and diacylglycerol which has diverse role as a second messenger and substrate in cells [10,17]. Distinct PERK-dependent mechanisms govern the response to acute and chronic ER stress. In contrast to the strict regulation of translation which occurs under transient stress, in response to chronic stress, PERK allows for a partial restoration in protein synthesis and simultaneous translation of UPR target genes. This switch allows cells to cope with chronic stress and evade cell death [18]. This may explain how many cancer cell-types (including breast tumours) exhibit constitutive PERK activation, and suggests that PERK may have roles in managing both acute insults experienced by cancer cells, and more prolonged stressors experienced within a tumour microenvironment such as hypoxia (See Section 3.2).

ATF6 is believed to primarily promote cell survival. Upon ER stress ATF6 translocates from the ER membrane to the Golgi where it is cleaved by site-1 and site-2 proteases. Cleaved ATF6 (ATF6f) then moves to the nucleus where it promotes expression of *XBP1*, *GRP78*, and ER-localized chaperones to promote protein folding and ER homeostasis (See Figure 2). Though ATF6 signaling predominantly promotes survival, it has also been linked indirectly to the downregulation of pro-survival BCL-2 family member myeloid cell leukemia 1 (MCL1) [10,19,20].

Prolonged and/or intense ER stress is lethal to normal cells, but in cancer UPR signaling is both sustained and non-lethal [21]. The current model of cell fate regulation by the UPR in normal cells consists of an early pro-adaptive response mediated by all three UPR arms that gives way to pro-death signaling which is believed to be primarily regulated by PERK/ATF4/CHOP and IRE1/JNK [6]. In contrast, chronic, non-lethal UPR signaling in breast cancer exhibits considerable heterogeneity in signaling output depending on the breast cancer subtype and the stressors experienced by cells. The next section will describe the evidence for altered expression, and mutations of UPR proteins in breast cancer and their roles in this disease.

## 3. Aberrant UPR Signaling in Breast Cancer

### 3.1. IRE1/XBP1s

Elevated levels of *IRE1* mRNA or protein do not necessarily imply IRE1 activation. Thus, XBP1s levels are commonly used as a readout of IRE1 activity. Notably, investigations of the role of IRE1 in breast cancer have focused exclusively on XBP1 and no data regarding roles for RIDD or IRE1 kinase activity have been reported. Unfortunately, probes which differentiate between the spliced and unspliced XBP1 isoforms are absent from most (if not all) high throughput gene arrays. Since the two XBP1 isoforms have different and even opposing functions [13], total XBP1 levels inform neither XBP1s activity nor IRE1 activation. To circumvent this limitation, researchers have begun examining XBP1s gene signatures (i.e., a set of genes known to be transcriptionally regulated by XBP1s) [9]. Immunohistochemical screens have also been hampered due to the lack of suitable antibodies specific to XBP1s or phosphorylated IRE1. Thus, older studies in which total XBP1 was used as a readout of IRE1 RNase activity should be interpreted cautiously.

A comprehensive study of gene expression signatures in primary samples revealed an overexpression of *XBP1* in luminal breast cancer, where it is co-expressed with *ESR1* [22]. Immunohistochemical analysis of 395 breast adenocarcinomas showed that 90% of samples stained strongly for XBP1 [23]. In a seminal paper, Laurie Glimcher’s group identified an XBP1 gene signature using ChIP-Seq which correlated with shorter relapse free survival in two cohorts of TNBC patients, but not in ESR+ breast cancer patients [9]. They also reported increased levels of XBP1 splicing in primary basal-like tumours compared to ER+/PGR+ tumours. These reports suggest that total XBP1 is overexpressed in luminal cancers while increased XBP1s transcriptional activity is more strongly associated with TNBC. This notion is corroborated in cell lines where basal-like cells are found to display higher levels of XBP1 splicing compared to luminal breast cancer and non-transformed cells [9,24].

Data mining using the Catalogue of Somatic Mutations in Cancer (COSMIC) platform revealed that IRE1 and XBP1 are rarely mutated in breast cancer (0.47% and 0.67%, respectively). However, IRE1 has been ranked as the fifth most likely kinase to harbor a driver mutation across other cancer types [25]. IRE1 mutations discovered in this study have been characterized in vitro and do not induce cell death when over expressed, unlike wildtype IRE1 which does [26]. In principle, this suggests that cancer cells can acquire mutations which prevent IRE1 from mediating cell death. Though no IRE1 mutations have been functionally characterized in breast cancer, using data from the COSMIC platform, we found nine base pair substitution mutations, five in the kinase domain and one silent mutation in the RNase domain (Table 1). The biological impact of these mutations is not known, although they do not occur at residues reported to be important for either IRE1 kinase or RNase activity.

XBP1 is highly expressed in luminal breast cancers but it is rarely found to be mutated [22]. However, complete genome sequencing of breast cancer and non-neoplastic tissue from 560 individuals revealed four possible exonic driver mutations in *XBP1*. The same study also reported seven mutations in the non-coding region surrounding the *XBP1* gene, at a rate significantly above that expected by chance [27].

Many in vivo and in vitro studies directly implicate XBP1 in the pathology of TNBC and luminal breast cancers. Using a transgenic mouse model where splicing of XBP1 produces a bioluminescence signal, it was found that mammary epithelial tumours displayed splicing of XBP1 throughout tumourigenesis [28]. In support of this finding, patient-derived BCM-2147 (TNBC), MDA-MB-231 (TNBC), NeuT EMTCL2 (mouse breast cancer cell line), and transformed MCF10A cells transplanted into mice form significantly fewer tumours when XBP1 was silenced [9]. A similar effect was observed with IRE1 knockdown [29]. Reciprocally, TNBC patient-derived cells exhibiting a non-stem cell-like phenotype (CD44-low/CD24-high) formed more tumours in mice when XBP1s was overexpressed [9]. In another study, knockdown of either IRE1 or XBP1 reduced angiogenesis in vivo [29]. Together, these studies show that XBP1 is important for TNBC tumour initiation and progression.

In vitro, XBP1s has been shown to interact directly with Hypoxia Inducible Factor 1α (HIF1α) [9], the key hypoxic stress-responsive transcription factor, and MYC proto-oncogene, bHLH transcription factor (MYC) [30]. The knockdown of XBP1 in TNBC cells caused a significant reduction in the expression of HIF1α target genes, such as vascular endothelial growth factor A (*VEGFA*), a key mediator of tumour angiogenesis. MYC was recently found not only to bind XBP1s, thereby potentiating XBP1s transcriptional activity, but also to bind to the promoter and enhancer region of *IRE1*, driving IRE1 expression and XBP1 splicing in TNBC [9,30]. Recent work from our laboratory has shown that IRE1 controls production and secretion of pro-inflammatory cytokines in TNBC cells. We showed that ablation of IRE1 RNase activity by small-molecule-mediated inhibition or by RNAi reduced cytokine secretion and shifted TNBC cells away from a stem cell-like phenotype [24]. These in vitro experiments have provided mechanistic insight into how IRE1 can become activated in TNBC and how XBP1 can drive tumour progression through direct interaction with other transcription factors, and through modulation of the tumour secretome [9,24,30].

**Figure 3:**
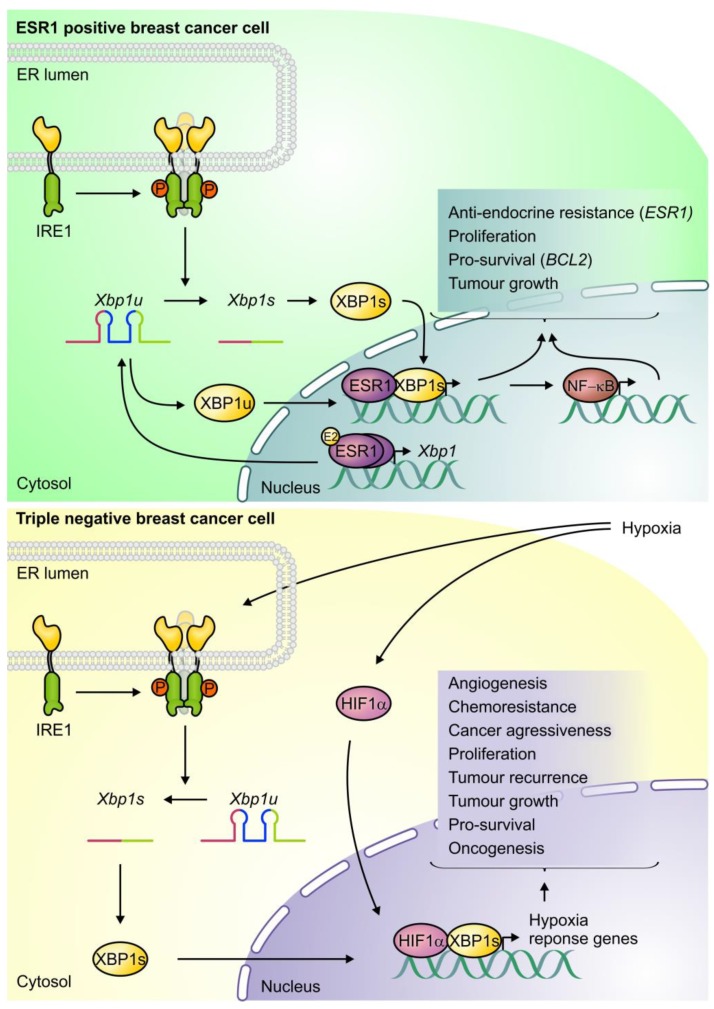
Dual roles of inositol-requiring enzyme 1 (IRE1)/X-box binding protein 1 (XBP1) in estrogen receptor 1 (ESR1)+ breast cancers and triple negative breast cancer (TNBC). Both XBP1 isoforms can activate ESR1 signaling in ESR1+ breast cancer cells and facilitate estrogen (E2)-independent tumour survival and proliferation. ESR1 signaling promotes expression of XBP1, thus generating a feed-forward mechanism. In TNBC cells, IRE1 exhibits high basal activity and activates XBP1s which dimerises with hypoxia inducible factor 1 subunit alpha (HIF1α) potentiating the expression of hypoxia response genes. This signaling drives tumour growth and angiogenesis (Lower panel adapted from Chen et al. [9]).

XBP1 promotes the growth of ESR1+ breast cancers by regulating ESR1 signaling. Estrogen drives many ESR1+ breast cancers and is an enduringly useful therapeutic target. Intriguingly, estrogen signaling activates all arms of the UPR in breast cancer cells both in vitro and in vivo [31,32]. XBP1 and ESR1 are co-expressed in luminal breast cancers and in vitro work has demonstrated the existence of a feed-forward mechanism connecting the two proteins [22,33]. Both XBP1s and XBP1u can trigger estrogen-independent ESR1 homodimerisation and transcription of ESR1 target genes [33], which include *XBP1* itself [34,35,36]. This allows ESR1+ tumours to achieve estrogen-independent growth and helps to explain why both XBP1 isoforms can drive ESR1+ cancer, but not TNBC. In support of this conclusion, a human luminal breast cancer cell line overexpressing either XBP1s or an unsplicable XBP1 mutant produced faster growing tumours when injected into mice compared to wildtype cells [36]. Other studies have demonstrated that lowering XBP1 levels in an ESR1+ cell line significantly reduced estrogen-stimulated growth [37]. Thus, IRE1/XBP1 signaling is intimately linked to ESR1 signaling in luminal breast cancer (see Figure 3).

### 3.2. PERK

Investigating PERK activity in high throughput datasets comes with caveats similar to IRE1. PERK mRNA and protein levels are not informative of PERK activity. Furthermore, high throughput transcriptomic analyses have limited utility since PERK targets such as ATF4 and CHOP are activated downstream of eIF2α phosphorylation, which can be mediated by three other kinases [15]. However, a PERK gene signature (determined by treating cells with PERK kinase inhibitor GSK2606414) has been correlated with a higher tumour grade and worse patient survival [38]. Elevated *ATF4* expression has been observed in breast cancer cells, both in vivo and in vitro [39,40], and high *CHOP* expression in a cohort of 250 breast cancer patients was associated with increased disease-free survival [41]. The only *bona fide* read-out of PERK activation is the level of phosphorylated PERK (p-PERK). In human breast ductal carcinoma in situ, p-PERK levels are increased compared with normal breast tissues [42] and p-PERK levels are higher in TNBC cell lines than in luminal cell lines [9]. COSMIC data mining revealed a very low PERK mutation rate in breast cancer (0.47%) and while five occur within the kinase domain none of the mutations occur at a residue with a known function (see Table 1). Breast tumour cells exploit PERK signaling to grow and to survive in harsh microenvironments. PERK ablation in Neu-driven mammary carcinoma cells and PERK knockdown in MDA-MB-468 (TNBC) cells led to smaller tumour volumes when injected into mice. Animals bearing *Perk*-null Neu-driven mammary tumours displayed increased tumour free survival compared to control mice. In a separate experiment the authors observed that aged mammary specific *Perk*-null mice spontaneously formed tumours compared to controls, suggesting that PERK has opposing roles in tumourigenesis [43].

Downstream of PERK, ATF4 mediates hypoxia-induced breast cancer progression via regulation of tribbles homolog 3 (TRIB3), unc-51-like autophagy activating kinase 1 (ULK1), and lysosomal-associated membrane protein 3 (LAMP3). All three genes are induced in hypoxic conditions via PERK/ATF4 and their knockdown, or knockdown of PERK and/or ATF4, reduces cancer cell proliferation (TRIB3 and ULK1), survival (ULK1), and migration (LAMP3) in hypoxia. Furthermore, higher TRIB3 and ULK1 expression is associated with a poor prognosis in breast cancer [44] while higher LAMP3 expression has been associated with lymph node positivity and hormone receptor negative breast cancers [45,46,47].

### 3.3. ATF6

ATF6 is activated through post-translational translocation and cleavage mechanisms [10] and there is no evidence that *ATF6* is transcriptionally upregulated in response to ER stress. Thus, presence of the ATF6f protein is the best readout for ATF6 activation. ATF6f gene signatures have been described for other cell types, but not for breast cancer. COSMIC analysis of breast cancers revealed just ten unique exonic ATF6 mutations in over two-thousand breast tumour samples (0.47%). Four mutations reside in domains important for the transcriptional activity of ATF6f, but none have been functionally characterized (Table 1).

Experimental evidence suggesting a direct role for ATF6 in breast cancer is limited. However, ATF6 knockdown was reported to reduce angiogenesis and tumour volume in a breast cancer xenograft model [29] and to significantly decrease estrogen-induced growth [32]. No molecular mechanism has been reported for these observations but ATF6 may play an indirect role through regulation of *XBP1* and *GRP78*.

### 3.4. GRP78

Elevated GRP78 levels are often taken as a readout of UPR activation since GRP78 is regulated by all three UPR arms; IRE1/XBP1s, PERK/ATF4 and AFT6f [48,49]. However, GRP78 is also a reported IRE1/RIDD substrate [50] and can be regulated in a UPR-independent manner [51,52,53,54,55,56]. Therefore, elevated GRP78 levels are at best only suggestive of UPR activity. Nonetheless, two independent tissue microarrays have shown GRP78 staining to be high in breast cancer with little or no positive staining observed in normal tissue [23]. GRP78 protein was found to be upregulated in primary HER2+ breast tumours when compared to HER2- tumours [57]. In vitro, GRP78 is more abundant in breast cancer cell lines compared to normal breast cells [58]. Further enrichment of GRP78 is found in stem cell-like subpopulations within multiple breast cancer cell lines [59].

In addition to the ER, GRP78 can be expressed in the cytosol and on the cell surface (sGRP78) where it may regulate transforming growth factor β (TGFβ) and phosphoinositide 3-kinase (PI3K) signaling [60,61]. sGRP78 has been detected on sub-populations of cells across different cancer types, including breast, but sGPR78 was either absent, or weakly expressed on normal cells. Expression of sGRP78 has been reported to increase following neoadjuvant treatment and is more abundant on hormone therapy resistant cells than on non-resistant cells [62].

Analysis of GRP78 mutations using the COSMIC platform revealed a low mutation rate (0.39%), similar to the UPR sensors. None of the mutations have been functionally characterized, however a frameshift mutation (p.A295fs*28) resulting in a stop codon occurs at a site reported to be important for ATP binding [63].

GRP78 has been demonstrated to promote breast tumour growth, angiogenesis and metastasis. Mammary specific oncogene-transformed Grp78^+/−^ mouse cells formed tumours less readily than Grp78^+/+^ cells. Grp78^+/−^ tumours were also significantly smaller and displayed lower microvessel density, indicating reduced angiogenesis [62]. GRP78 knockdown blocked lung metastasis in a xenograft model using human TNBC cells [64], while overexpression increased metastasis [65]. In vitro, overexpression of GRP78 increased the migration and invasion of breast cancer cells while GRP78 knockdown reduced it [64]. These studies provide a strong rationale for targeting GRP78 in breast cancer.

Mutations in XBP1, IRE1, PERK, ATF6, and GRP78 were compiled using COSMIC database (https://cancer.sanger.ac.uk/cosmic) [66]. “Breast Cancer” was used as the search term on the home page. “breast, carcinoma” was selected as the disease type. Under the “Genes” heading the “Genes with mutations” tab was selected. Searches were performed for XBP1, “ERN1” (IRE1), “EIF2AK3” (PERK), “ATF6” and “HSPA5” (GRP78). The “Variants” tab was selected, and the variants were exported to excel in “CSV” format. The domains in which the variants occurred was manually annotated with reference to UniProt (https://www.uniprot.org/). The interrogation for this review was performed on 28 July 2018. The database may have been updated since then.

## 4. UPR Signalling Promotes Therapy Resistance in Breast Cancer

The UPR is reported to confer breast cancer cells with resistance to radiation therapy [67], tamoxifen [36], paclitaxel, vinca alkaloids [68], cisplatin [69], doxorubicin [9], histone deacetylase (HDAC) inhibitors [62], and microtubule interfering agents [70]. Conversely, the UPR can promote breast cancer cell death in response to other therapies such as bortezomib [71], lapatinib/obatoclax combination [72] and pan-peptidylarginase deiminase [73]. These conflicting findings highlight the context-specific nature of UPR output, and the need to understand the contribution of each UPR arm individually. Since the severity of ER stress is a determinant of cell fate, it is plausible that the therapeutic dose achieved in tumour cells is also a determining factor on whether the UPR promotes survival or death rather than the drugs mechanism of action.

### 4.1. IRE1/XBP1

IRE1/XBP1 has been shown to confer resistance to doxorubicin and paclitaxel in TNBC and to tamoxifen in ESR1+ cancers [9,36]. Human TNBC cells injected into mice developed resistance to doxorubicin and paclitaxel treatment over time but XBP1 knockdown prevented tumour recurrence [9]. In a separate study, overexpression of XBP1s in ESR1+ cells led to tamoxifen resistance in vivo. Mice injected with cells bearing a more stable and unsplicable XBP1u were also resistant to tamoxifen, but to a lesser extent [36]. In vitro, XBP1s overexpression in an ESR1+ breast cancer cell line increased levels of the pro-survival protein BCL-2 and decreased mitochondrial membrane permeabilization when cells were challenged with the estrogen antagonist’s tamoxifen or fulvestrant [74]. Other in vitro work has shown that in ESR1+ cells both XBP1 isoforms contribute to tamoxifen resistance via NF-κB [36] (See Figure 3).

### 4.2. PERK

PERK can promote resistance to paclitaxel, doxorubicin, and radiation in breast cancer but is required for drug-induced cell death in some circumstances. PERK becomes active in human mammary epithelial (HMLE) cells induced to differentiate, and phosphorylates NRF2 thereby reducing cellular ROS and promoting resistance to paclitaxel and doxorubicin [38]. Radiation treatment of breast cancer cells induces the PERK/ATF4/LAMP3 pathway. Knockdown of this pathway reduces the DNA damage response in breast cancer cells and sensitizes them to radiation-induced death [75].

Two studies have demonstrated a role for PERK in promoting breast cancer cell death in response to drug treatment, but reveal divergent outcomes of PERK-regulated autophagy on cell viability. Knockdown of PERK promotes survival of luminal breast cancer cells treated with a combination of lapatinib (a tyrosine kinase inhibitor) and obatoclax (a pro-survival BCL-2 family inhibitor) by reducing pro-death autophagy [73]. However, the PERK-ATF4 pathway was shown to be crucial for the ability of a pan-peptidylarginase deiminase to kill TNBC cells in vitro and reduce tumour growth in vivo through activation of mTOR signalling and perturbation of autophagy [72]. At the core of this discord is the dual-role of PERK in promoting and inhibiting autophagy. Therefore, we should be cautious when targeting PERK in combination with other drugs and proceed on a contextual basis.

### 4.3. ATF6

There is no direct evidence that ATF6 plays a role in drug-resistance, although one study found ATF6 to be an independent predictor of increased relapse and shorter survival in primary ESR1+ breast cancers treated with tamoxifen [32].

### 4.4. GRP78

GRP78 confers breast cancer cells with resistance to radiation, anti-hormonal therapy, combretastatin A4P (anti-vascular agent) contortrostatin (anti-angiogenic agent) [76], microtubule-interfering agents [70], HDAC inhibitors, vinca alkaloids [70], and gemcitabine [77]. Xenograft experiments have shown that tamoxifen induces GRP78 expression in breast tumours, and when GRP78 is silenced, response to tamoxifen is restored [78]. In vitro, knockdown of GRP78 sensitizes ESR1+ breast cancer cells to paclitaxel, vinblastine [70], etoposide, and radiation while GRP78 overexpression confers resistance to gemcitabine [77]. Thus, GRP78 appears to confer breast cancer cells with resistance to multiple drugs [62].

## 5. UPR-Targeting Drugs: Stand-Alone and Combination Therapies

Many compounds are available which target UPR proteins (see Table 2), although none are approved for use in patients [79,80]. However, synergistic anti-breast tumour effects have been observed in the few studies that have investigated the combination of FDA-approved chemotherapeutic drugs and pre-clinical UPR-targeting agents. Since therapy-resistance is a leading cause of tumour recurrence and patient mortality, these reports constitute a promising solution to an urgent clinical challenge.

Several IRE1 RNase inhibitors that block XBP1 splicing and RIDD; MKC3946, 3-methoxy-6-bromosalicylaldehyde, 4μ8C, STF-083010, and Toyocamycin, have shown promise in multiple myeloma models, where XBP1s is known to be important for tumour progression [80]. More recently, an IRE1 RNase inhibitor MKC8866 was shown to reduce the growth of patient derived xenograft breast tumours when administered alone [30]. However, another study using a MDA-MB-231 xenograft model showed that MKC8866 had no effect on tumour growth as a stand-alone treatment [24]. Intriguingly, the genotoxic drug doxorubicin was recently identified as a potent inhibitor of the IRE1 RNase [81] which has presumably contributed to its efficacy in some cases. In vivo, STF-083010 significantly reduced tamoxifen resistant ESR1+ tumour growth both as a stand-alone therapy and in combination with tamoxifen [82]. Recently, a combination treatment of MKC8866 and docetaxel was shown to eliminate MYC driven tumours in a patient-derived xenograft model [30]. Syngeneic mouse models revealed the same effect on tumour growth in mice with an intact immune system, though only in MYC-driven tumours [30]. Recent work from our laboratory has shown that paclitaxel induces XBP1 splicing in TNBC and that MKC8866 significantly sensitizes TNBC tumours to paclitaxel in a murine xenograft model [24].

There are three ATP-competitive PERK kinase inhibitors, GSK2606414, GSK2656157 and AMG PERK 44 [83,84]. GSK260414 has been shown to reduce metastasis of breast cancer cells in vivo [85]. However, GSK2606414 and GSK2656157 also inhibit receptor interacting serine/threonine kinase 1 (RIPK1, a regulator of cell death and inflammation) while AMG PERK 44 does not [86]. Indeed, AMG PERK 44 is over 160 times more selective for PERK compared with 387 kinases tested, though it has not been tested in breast cancer models [84]. GSK2606414 has been shown to sensitize de-differentiated HMLEs to paclitaxel and doxorubicin in vitro, and to reduce xenograft tumour growth of TNBC cells in the presence of doxorubicin [38]. A very recent study identified novel inhibitors of the ATF6 pathway, though their mechanism of action was not determined [87].

There are several pre-clinical agents, which reduce the activity of GRP78 though only VER-155008 [63] and HA15 [88] are reported as direct binders [62]. An anti-GRP78 scFv has been shown to reduce breast tumour growth in xenograft models [89]. Pharmacological targeting of sGRP78 with a monoclonal antibody (MAb159) reduced growth and metastasis of TNBC cells in vivo and GRP78-targeting peptide (BMTP78) reduced growth of pre-established bone micrometastases in a TNBC xenograft and syngeneic murine models [62]. An anti-GRP78 antibody PAT-SM6 has gone through phase I trials for myeloma [90] (Trial ID: NCT01727778) and melanoma (ACTRN12610000733077) and appears to be well tolerated [62]. Another compound, NKP-1339, which reduces GRP78 levels [91] through an unknown mechanism, is also in clinical trials for solid tumours (Trial ID: NCT01415297). In vitro, Plumbagin, a compound which lowers GRP78 levels, sensitized breast cancer cells to tamoxifen [92].

## 6. Future Perspectives and Challenges

The role of the UPR in breast cancer remains to be fully elucidated. Given that the UPR consists of three distinct, yet interdependent, signalling pathways, each with a myriad of reported functions, deciphering the precise mechanisms through which the UPR is co-opted to promote cancer in a given context poses a significant challenge. For example, the divergent function of the IRE1 RNase domain is emerging as a particular barrier to our understanding of the UPR in cancer. The RIDD activity of IRE1 is perhaps the least studied aspect of UPR biology and no roles for RIDD have been reported in breast cancer. Since IRE1/RIDD is reported to promote cell death [6] it may function as a tumour suppressor as opposed to XBP1s which is pro-tumour. In fact, XBP1 splicing and RIDD have recently been shown to have opposing functions in glioblastoma [127], a discovery which complicates the targeting of IRE1 in the clinic. Since RIDD has not been studied in breast cancer, it remains a completely open question as to whether targeting global IRE1 RNase activity would be preferable to targeting XBP1 splicing alone in this context. As such, approaches that block XBP1s, while maintaining RIDD, like targeting RNA 2′,3′-Cyclic Phosphate and 5′-OH Ligase (also known as RTCB, the enzyme which ligates spliced XBP1) [128] for example, may prove more effective than IRE1 RNase inhibition in some circumstances. RTCB inhibition may also be preferable to IRE1 RNase inhibition in luminal breasts cancers, since in addition to reducing XBP1s levels, total XBP1 levels should, in theory, also be reduced. Defining the molecular determinants which govern whether IRE1 RNase engages XBP1 splicing or RIDD activity is an active area of research for the UPR field [129], and is likely to yield insights which may eventually allow both activities to be individually modulated in patients.

The UPR is emerging as a pathway that not only rewires cancer cells but also influences the tumour microenvironment. The release of pro-inflammatory cytokines and chemokines from cancer cells is known to influence the tumour microenvironment resulting in the recruitment of immune cells which can promote angiogenesis, invasion, and metastasis [130]. Given the emerging importance of IRE1/XBP1s signalling in inflammation it is plausible that sustained IRE1 activity may influence release of inflammatory mediators, which in turn could impact the tumour microenvironment. Indeed, culture of naive cells with conditioned media from cells challenged with ER stress triggers pro-inflammatory cytokine production in recipient cells [131]. This phenomenon, called “transmissible ER stress” (TERS), suggests that UPR activation in tumour cells may elicit pro-tumourigenic effects distinct from the classical role of UPR. In fact, a study from 2013 found that addition of media from an ER stress treated mouse breast cancer cell line was able to induce a pro-inflammatory phenotype in recipient macrophage cell line and increased expression of the pro-angiogenic molecule VEGF. Recent work from our laboratory has found that IRE1 controls the production and secretion of IL6, IL8, CXCL1 and GMCSF in TNBC cells. Since all of these factors play a role in modulating the function of the immune system [132]. These results highlight the potential of targeting the UPR to repress tumour growth via modulation of the tumour microenvironment.

Targeting the UPR in the clinic poses several challenges, including the possibly of unwanted side-effects. Given the diversity of UPR functions, how confident can we be that UPR inhibition will not create more problems than it solves? For instance, XBP1 is required for the differentiation of B-cells into plasma cells [133], the differentiation of CD8^+^ T-cells during infection [134], the development and function of dendritic cells [135,136,137], the expression cytokines after TLR stimulation in macrophages [138], eosinophil differentiation [139], and the function of intestinal Paneth cells which secrete anti-microbial proteins [140,141]. This makes predicting the outcome of UPR inhibition difficult. However, using a syngeneic model of breast cancer Zhao et al. have recently demonstrated that MKC8866 can reduce tumour growth when administered with docetaxel to mice with a competent immune system. Furthermore, the authors observed increased CD4^+^ and CD8^+^ immune cell infiltration in tumours that received MKC8866 and docetaxel [30]. This suggests, at least in this context, that whatever effect IRE1 RNase inhibition is having on cell types throughout the organism, the net result is a reduction in tumour burden and enhanced anti-tumour immune cell activity. Furthermore, long-term administration of MKC8866 did not cause any damage to the pancreas of the animals, an organ which relies on the UPR for its secretory function. This promising report suggests that IRE1 RNase inhibition may promote immune infiltration and tumour cell destruction without any apparent side-effect, but will this finding hold true when UPR inhibition is investigated in other cancer types? In a model of ovarian cancer, XBP1 was found to drive a pro-tumour mechanism in dendritic cells, suggesting that targeting this branch of the UPR may promote increased tumour cell killing by dendritic cells [137]. Despite these reports, more studies are required to rule out the potential for unwanted side-effects when targeting the UPR. A crucial question the field faces is: how can we determine when patients will benefit from UPR-targeting drugs and when should such drugs be avoided? This would appear to necessitate biopsy of the tumour and determination of which UPR arms are active in the tumour cells. Addition of UPR markers to routine assays used to subtype breast tumours could be a useful way to obtain this information. The discovery of TERS hints that biomarkers of specific disease states may be released by diseased cells experiencing ER stress. This suggests that a less invasive method could be employed as a determinant of UPR activation, and perhaps even an aid to diagnosis. A lot of research remains to be done before the utility of using UPR-associated secreted molecules as disease-specific biomarkers is properly determined. For now, it remains an exciting future prospect.

## 7. Conclusions

In conclusion, UPR signalling is active in breast cancer, promoting the development and progression of the disease, and contributing to therapy resistance. Basic research has provided compelling evidence is support of targeting IRE1/XBP1, PERK, and GRP78 to improve treatment outcome for breast cancer in the clinic. Inhibitors of these proteins have been developed and shown to reduce the growth of breast tumours in vivo both alone and in combination with FDA-approved drugs to which the UPR confers resistance. The role of the UPR in breast cancer is subtype-dependent which makes the UPR a challenging therapeutic target. However, the specifics of UPR signalling in breast cancer are becoming increasingly clear which will allow anti-UPR drugs to be tailored to specific breast cancers and to specific drugs in combination therapies.

## Figures and Tables

**Table 1:** Catalog of Somatic Mutations in Cancer (COSMIC) Database Interrogation for Unfolded Protein Response (UPR) Mutants.

**Inositol-requiring enzyme (IRE1)**	
Luminal domain	p.P75Q, p.A371A, p.H386fs*8
Transmembrane domain	p.L454L
Cytoplasmic domain	p.Q495_L496insQ
Kinase domain	p.G703D, p.L714L, p.V767A, p.R806C, p.A823V, p.F937F
**X-box binding protein 1 (XBP1)**	
bZIP/nuclear localization signal	p.R81fs*16, p.R90P
bZIP/leucine zipper	p.E108delE, p.E121D
Translational pausing of own mRNA	p.L236fs*16, p.L238fs*13
Other regions	p.P8P, p.P37A, p.Q43E, p.E97delE, p.S187fs*6, p.S190fs*1, p.P213fs*45, p.L232fs*22
**PKR-like ER Kinase (PERK)**	
Luminal domain	p.R114I, p.S385R
Cytoplasmic domain	p.T537T, p.R588P, p.D1081fs*31, p.L1088L, p.S1098L
Cytoplasmic/kinase domain	p.S686F, p.C788C, p.R797T, p.R1027G, p.E1050D
**Activating transcription factor 6 (ATF6)**	
Cytoplasmic/transcription activation	p.E25Q
Cytoplasmic domain	p.Q237 *
Cytoplasmic/basic motif	p.R309K, p.K327N,
Cytoplasmic/bZIP	p.E365Q
Luminal domain	p.A450fs*7, p.C467fs*1, p.L477F, p.R484Q, p.S592S, p.R624S, p.S631L
**Glucose-regulated protein 78 kDa (GRP78)**	
Signal peptide	p.L13L
Nucleotide-binding domain	p.I132T, p.K138N, p.T166T, p.E243K
ATP-binding	p.A295fs*28
Other regions	p.E308Q, p.E514Q, p.E603E

**Table 2:** UPR Targeting Drugs and their site of action.

**Inositol-Requiring Enzyme 1 (IRE1)**	
RNase domain inhibition	Toyocamycin [93], MKC3946 [94], 4μ8c [95], 3-Methoxy-6-bromosalicyl-aldehyde [96], STF083010 [97], Doxorubicin [81], MKC8866 [24,30], B-H09 [98], 2-hydroxy-1-naphthaldehyde [99]
Q-site	Quercetin [100]
Kinase domain inhibition	APY29 [26], Sunitinib [101], Compound 3 [102], KIRA6 [26], KIRA8 [103], UPRM8 [104], GSK2850163 [105], FIRE [106]
Not determined	Resveratrol [107], 3,6-DMAD [108]
**PKR-like ER Kinase (PERK)**	
Kinase inhibition	GSK2606414 [109], GSK2656157 [110], AMG PERK 44 [84]
Kinase activation	Compounds A, B, C [111], DHBDC [112]
Inhibit downstream effect of EIF2A	ISRIB [113]
Promotes maintenance of EIF2A phosphorylation	Salubrinal [114], Guanlabenz [115]
**Activating transcription factor 6 (ATF6)**	
Inhibit nuclear translocation	CEAPIN Class 1 [87]
Inhibit transcriptional activity	CEAPIN Class 2 [87],
PDI inhibitor	PACMA 31 [116], RB11-ca [117], P1 [118], 16F16 [119]
Prevent AFT6 cleavage (Serine protease inhibitor)	AEBSF [120]
Not determined	Melatonin [121], Compounds 147, 263 [122]
**Glucose-regulated protein 78 kDa (GRP78)**	
Reduce GRP78 levels	OSU-03012 (AR-12) [123], Deoxyverrucosidin [124] Plumbagin [92], HA15 [88], DHA [125],
Inhibit GRP78 activity	PAT-SM6 [90]
Block GRP78 transcriptional induction	Arctigenin [126]
